# Comparison of Patients’ and Surgeons’ Expectations before Shoulder Arthroplasty

**DOI:** 10.3390/jcm13123489

**Published:** 2024-06-14

**Authors:** Katrin Karpinski, Fabian Plachel, Christian Gerhardt, Tim Saier, Mark Tauber, Alexander Auffarth, Alp Paksoy, Doruk Akgün, Philipp Moroder

**Affiliations:** 1 Centrum für Muskuloskeletale Chirurgie, Klinik für Schulter- und Ellenbogenchirurgie, Charité Universitätsmedizin Berlin, 13353 Berlin, Germanyalp.paksoy@charite.de (A.P.); moroder.info@kws.ch (P.M.); 2 St. Vincentius-Klinik Karlsruhe, 76137 Karlsruhe, Germany; christian.gerhardt@vincentius-ka.de; 3 BG Unfallklinik Murnau, 82418 Murnau am Staffelsee, Germany; 4 ATOS Klinik München, 81925 München, Germany; 5 Universitätsklinik für Orthopädie und Traumatologie, Universitätsklinikum Salzburg, A-5020 Salzburg, Austria; a.auffarth@salk.at

**Keywords:** shoulder arthroplasty, patients expectations

## Abstract

**Background:** Patients suffering from osteoarthritis particularly complain about pain during day and night as well as loss of function. This consequently leads to impaired quality of life and therefore psychological stress. The surgical therapy of choice is joint replacement. Regarding the outcome after operation, expectations might differ between the patient and the surgeon. This can lead to dissatisfaction on both sides. This study aimed to document patients’ expectations of a planned shoulder joint replacement. The results were compared with assessments made by shoulder surgeons. **Methods:** In total, 50 patients scheduled for operative shoulder joint replacement were included in this study, as well as 10 shoulder surgeons. Patients were requested to fill out questionnaires preoperatively to provide sociodemographic data, PROMS (Patient-Reported Outcome Measures) with regard to the pathology and their expectations about surgery in terms of pain relief, gain of range of motion, strength as well as the impact on activities of daily and professional life and sports. In addition, surgeons were asked what they thought their patients expect. **Results:** The most important goal to achieve for patients was to relieve daytime pain, followed by improvement of self-care and the ability to reach above shoulder level. The most important factors for patients to achieve after operation were ‘pain relief’ in first place, ‘movement’ in second and ‘strength’ in third. This also applied to shoulder surgeons, who ranked ‘pain relief’ first, followed by ‘movement’ and ‘strength’. When patients where asked what is most important when it comes to choosing their surgeon, 68% voted for ‘surgical skills’, 28% for ‘age/experience’, followed by ‘empathy’, ‘sympathy’ and ‘appearance’. For surgeons, ‘age/experience’ obtained rank one, ‘surgical skills’ was ranked second, followed by ‘sympathy’, ‘empathy’ and ‘appearance’. Surgeons significantly underrated the factor ‘empathy’ in favor of ‘sympathy’. **Conclusions:** This study shows that patients’ expectations for shoulder joint replacement and surgeons’ assessments do not differ significantly. Relief from pain and better shoulder movement were crucial for patients to achieve after operation, which was in line with surgeons’ expectations. The most important factor for choosing the surgeon was ‘surgical skills’ for patients, while surgeons thought they would care more about ‘age and experience’. This underlines that patients’ expectations should be taken into account within the preoperative medical interview. This might allow an optimization of compliance of the patients and lead to a better satisfaction on both sides.

## 1. Introduction

Osteoarthritis of the shoulder can be divided into primary osteoarthritis, which mostly affects patients over 60 and is related to sex, body mass index (BMI) and physical activity, and secondary osteoarthritis, which often occurs due to a large rotator cuff lesion or after a fracture which leads to changed shoulder kinematics [1,2]. Patients especially suffer from pain during both day and night and loss of range of motion, which impairs activities of daily and professional life. Consequently, their quality of life is significantly affected.

Different therapies are at surgeons’ disposal, including conservative and operative procedures. The decision is mostly based on the severeness of osteoarthritis, the patient’s subjective symptoms as well as comorbidities and psychological stress. The current ‘gold standard’ for treating osteoarthritis operatively is arthroplasty. Several studies show significant improvement with regard to pain, motion and various scores in the short, medium and long term [3,4,5]. 

As symptoms differ preoperatively, patients will present with various demands regarding joint replacement. It is known that patients’ expectations might influence the outcome after surgery [6]. When expectations of patients and surgeons are divergent, it may lead to dissatisfaction and frustration for both [7].

The aim of this study was to analyze patients’ and surgeons’ expectations regarding a planned shoulder joint replacement surgery with standardized questions and compare the results. Furthermore, the impact of different parameters on the patients’ expectations was calculated. 

## 2. Materials and Methods

Four hospitals in Germany and Austria specialized in shoulder surgery participated in this study. In total, 50 patients scheduled for operative shoulder joint replacement were consecutively included from August 2018 to October 2019. Furthermore, 10 senior physicians who specialized in shoulder surgery and who worked at the involved institutions were interviewed about their expectations.

Before being scheduled for surgery, patients presented during consultation hours to discuss conservative and operative options for their underlying pathology. When the decision for operative treatment was made, patients received a questionnaire with three parts before undergoing surgery. The first included sociodemographic data (A), the second was a validated patient-reported outcome questionnaire with regard to the underlying pathology (B), and the third part inquired about their expectations concerning the planned operative treatment (C).

Patients independently filled in form A. The first part asked about socio-demographic data such as relationship status, education and insurance. 

Part B assessed the underlying pathology and the impairment on daily life. It consisted of the American Shoulder and Elbow Surgeons Score (ASES) and the Subjective Shoulder Value (SSV). The ASES is a self-assessment tool to capture the patient’s activities of daily life. The maximum score is 100 points, which indicates no restrictions of activities of daily life [8]. With the SSV, patients can express how much their shoulder is affected as a percentage of a healthy shoulder, which scores 100% [9]. Furthermore, Part B included a Visual Analog Scale (VAS) for pain and investigated the etiology of the pathology as well as preceding therapy [10].

Part C investigated expectations for pain, range of motion and strength. Additionally, the included Shoulder Surgery Expectations Survey (SSES) examined the expected effects on activities of daily and professional life and sports [11]. Patients were asked to rank the parameters ‘strength’, ‘pain relief’, ‘cosmetics’, ‘movement’ and ‘stability’ in order of importance to achieve after operation. Every parameter was ranked regarding the factor ‘pain relief’. Furthermore, patients were queried as to what they would accept with regard to pain, duration of immobilization, physiotherapy, scars and aftercare following surgery. When choosing their surgeon, patients were asked what parameter (surgical skills, age/experience, sympathy, empathy, appearance) was the most important to them. 

All surgeons were asked to answer part C of the questionnaire, analyzing their assessment of the patients’ expectations regarding shoulder arthroplasty. 

### Statistical Analysis

The Kolmogorov–Smirnov test was used to test all data for normal distribution. Statistical analysis was conducted with the SPSS software version 27 (IBM-SPSS, New York, NY, USA). To determine the difference between patients’ and surgeons’ expectations, the Mann–Whitney U test and the Pearson correlation coefficient were calculated. Spearman’s correlation coefficient was used to identify correlations between sociodemographic data and patients’ expectations. A *p* value of less than 0.05 (* *p* ≤ 0.05, ** *p* ≤ 0.01) indicated statistical significance.

## 3. Results

### 3.1. Patient Demographics

At the time of surgery, the mean age of patients was 71.6 ± 11.7 years (range 33–91). Among the patients, 34 (68%) were female and 16 (32%) were male. The average BMI was 28.0 ± 6.0 (range 18.2–43.2). In 28 cases (56%), the dominant side was affected. Regarding insurance, 36 patients (72%) were compulsorily insured, and 14 (28%) had private insurance. Ten patients (20%) were single, 4 (8%) were in a relationship, 29 (58%) were married, 6 (12%) were divorced, and 1 patient (2%) was widowed. For their highest education level, 21 patients (42%) selected ‘apprenticeship’, 9 patients (18%) indicated ‘high school graduation’, 4 patients (8%) had a ‘bachelor’ degree, 4 patients (8%) had a ‘master’, 2 patients (4%) had a ‘doctorate’, and 10 patients (20%) responded with ‘other’. Asked about how much they care about health, 19 patients (38%) answered with ‘a lot’, 26 patients (52%) with ‘reasonable’ and 5 patients (10%) with ‘moderate’.

### 3.2. Preoperative Status

The average value of the ASES score was 35.8 ± 8.1 (range 13–50). The mean SSV was 27.4 ± 17 (range 0–60). The preoperative VAS scored a mean of 6.6 ± 2 (range 2–10). When asked about the reason of their osteoarthritis, 7 (14%) answered with ‘sports injury’, 2 (4%) with ‘work-related accident’, 13 (26%) with ‘other accident’ and 28 (56%) with ‘spontaneous/over time’. Among the patients, 40 (80%) had already undergone therapy; of these, 17 (42.5%) had surgery, 12 (30%) had physiotherapy, 3 (7.5%) received ‘other’ therapy (e.g., infiltration), and 8 (20%) indicated a combination. Patients reported an average length of their symptoms of 56 ± 64 months (range 1–360).

### 3.3. Patients’ and Surgeons’ Expectations

**Figure 1:**
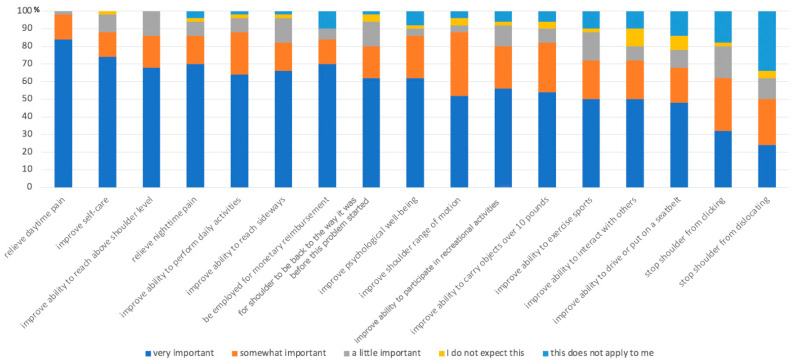
Patients’ expectations using the‚ Hospital for Special Surgery Shoulder Surgery Expectations Survey’.

The most important objective for patients undergoing shoulder arthroplasty was to relieve daytime pain (‘very important’ for 84%, ‘somewhat important’ for 14%). The second most important was to improve self-care (‘very important’ for 74%, ‘somewhat important’ for 14%). The ability to be able to reach above shoulder level was third (‘very important’ for 68%, ‘somewhat important’ for 18%). A detailed evaluation of the SSES is provided in Figure 1. 

**Figure 2:**
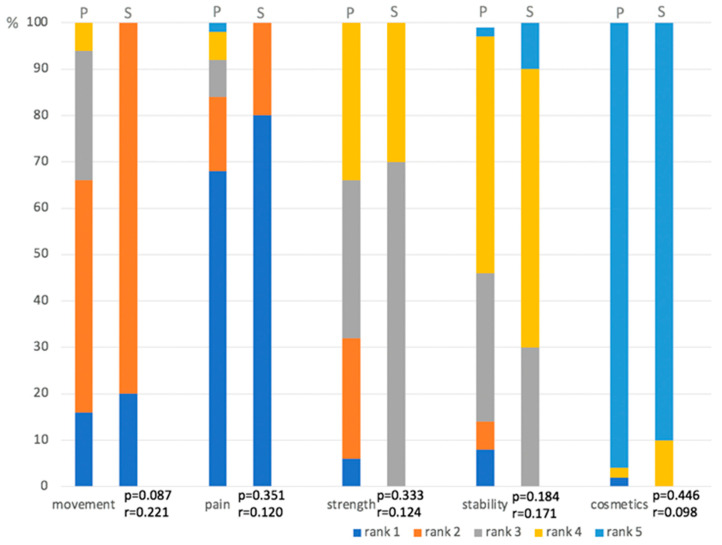
Patients’ (P) and surgeons’ (S) ranking of the factors movement, pain, strength, stability and cosmetics being the most important to achieve after shoulder arthroplasty.

When asked which factor was the most important for patients to achieve after surgery, the first place was ‘pain reduction’ (68% rank 1, 16% rank 2, 8% rank 3), the second was ‘movement’ (16% rank 1, 50% rank 2, 28% rank 3), and the third was ‘strength’ (6% rank 1, 26% rank 2, 34% rank 3). For shoulder surgeons, the most important factors were ‘pain reduction’ (80% rank 1, 20% rank 2), second was ‘movement’ (20% rank 1, 80% rank 2), and third was ‘strength’ (70% rank 3, 30% rank 4). The ranking is displayed in Figure 2. 

**Figure 3:**
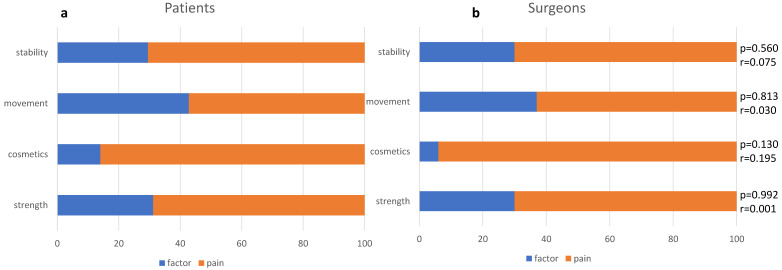
Patients’ (**a**) and surgeons’ (**b**) ratio of importance between pain and other outcome parameters.

The results of ranking those parameters against ‘pain’ are shown in Figure 3. 

**Figure 4:**
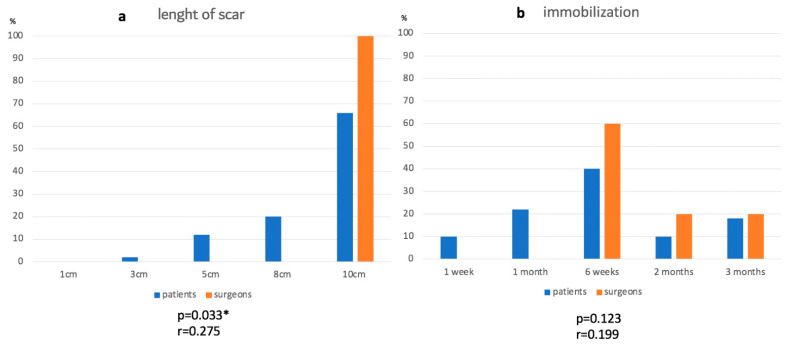

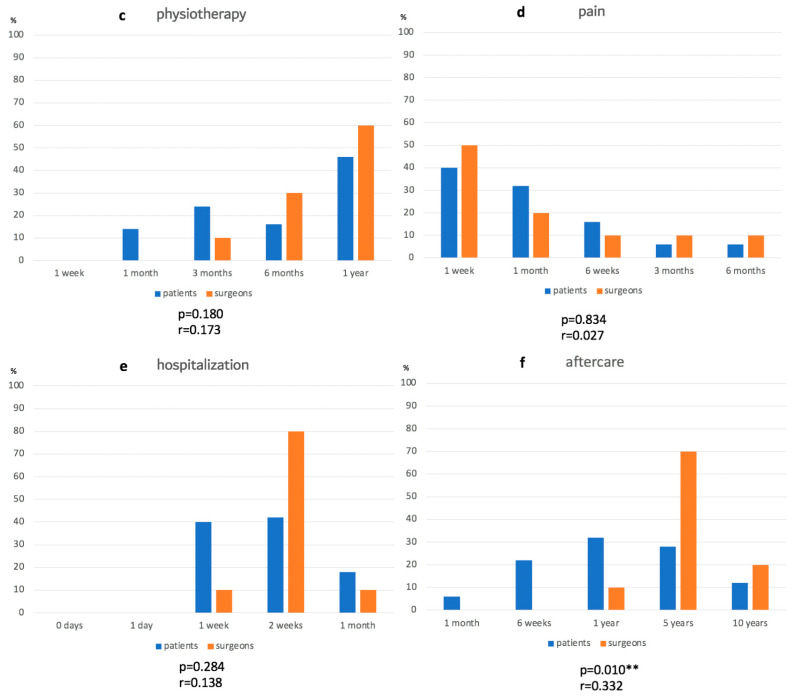
Patients’ acceptance for 100% shoulder function and surgeons’ anticipation. (**a**) length of scar, (**b**) immobilization, (**c**) physiotherapy, (**d**) pain, (**e**) hospitalization, (**f**) aftercare. * *p* ≤ 0.05, ** *p* ≤ 0.01.

Figure 4a–f display what patients would accept in order to gain a shoulder that is 100% working and pain-free in terms of scars, immobilization, physiotherapy, postoperative pain, hospitalization and aftercare. 

With regard to choosing the surgeon, the most important factor for patients was ‘surgical skills’ (68%) and ‘age/experience’ (28%), followed by ‘empathy’, ‘sympathy’ and ‘appearance’. For surgeons, ‘age/experience’ obtained rank 1 and ‘surgical skills’ was rank 2, followed by ‘sympathy’, ‘empathy’ and ‘appearance’, which ranked last (Figure 4a–e). Surgeons significantly underrated the factor ‘empathy’ (*p* = 0.034), which was more important for patients than ‘sympathy’. 

The influence of patients’ demographics and preoperative scores on the expectations is displayed in Table 1.

## 4. Discussion

The current study showed that no fundamental gap exists between the subjective expectations of patients and surgeons’ expectations with regard to the outcome after shoulder joint replacement. The most important goal to achieve for patients suffering from osteoarthritis was ‘to relieve daytime pain’, followed by ‘to improve self-care’ and ‘the ability to be able to reach above shoulder level’, which is in line with a recently published study [12]. Patients and shoulder surgeons agreed that pain relief is the most important goal after surgery, followed by an optimization of shoulder movement and increased strength.

Nevertheless, surgeons significantly overestimated the postoperative aftercare patients would undertake (Figure 4f). While 70% of surgeons thought 5 years and 20% thought 10 years would be acceptable, 32% of patients preferred aftercare of 1 year, 28% of 5 years and 22% of 6 weeks. This might be explained by the fact that patients already had a long medical history, and one goal of the operation is to finally remove the need to visit a doctor. This underlines that aftercare and its status for the postoperative outcome should also clearly be discussed with the patient.

With regard to postoperative pain, most of the surgeons and patients answered ‘one week’. This is quite plausible, as getting rid of pain is one major goal of shoulder replacement surgery.

**Figure 5:**
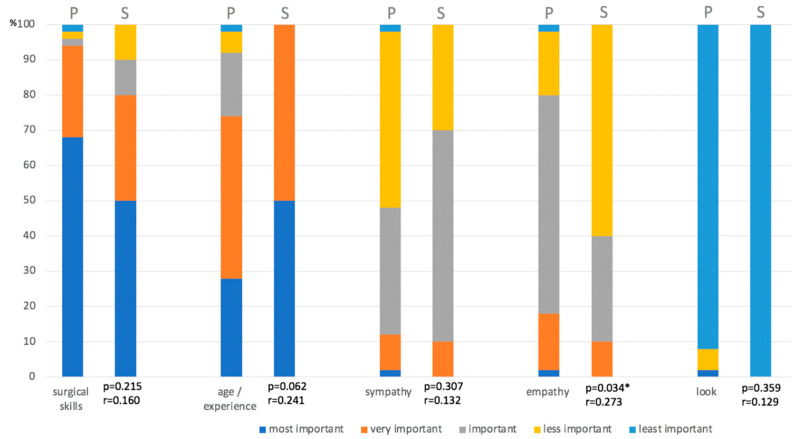
Opinion about the most important factor for choosing one‘s surgeon (P = patients, S = surgeons). * *p* < 0.05.

When it comes to choosing the surgeon, ‘surgical skills’ was the most important factor for patients, while surgeons thought ‘age and experience’ would be the most crucial. This shows that patients would also trust a younger surgeon, if they were aware of his or her skills. The factor ‘empathy’ was more important for patients than what surgeons estimated, underlining the need not only to assure the patient with one’s skills but also showing awareness of the patient’s needs and fears (Figure 5).

When discussing therapy options with their patients, shoulder surgeons should take into account that the improvement of movement of the shoulder was especially important for patients with a lower ASES, as some procedures might not improve the individual postoperative range of motion.

There was a correlation between higher age and importance of participating in sports activities again, which initially seems to be counterintuitive; however, this result could be explained by the fact that a longer history of shoulder pain in older patients prevented them from participating in sports as an important source of socializing for a long time.

The higher the SSV and the lower the VAS were preoperatively, the more important it was for the patient to relieve nighttime pain. As nighttime pain is one of the first symptoms of osteoarthritis, this correlation might be explained by assuming that patients having better preoperative scores did not suffer too long from their symptoms. This also applies to younger patients that would accept a longer period of immobilization and aftercare as well as postoperative pain.

The longer patients suffered from their symptoms, the more important a likeable surgeon was for them, probably based on a long medical history with visits to various doctors. As the same applies to patients with a high SSV preoperatively, surgeons should also rely on their sympathy in addition to their surgical skills, as this is one of the first attributes patients get to know during consultation to build up a trustful relationship.

Greater preoperative expectations go hand in hand with better self-assessed outcome of patients as already suggested by previous studies [13,14,15,16,17,18,19,20,21,22]. It can be derived from this fact that a patient’s expectation itself is an independent outcome predictor amongst other variables such as age and comorbidities, extent of osteoarthritis or duration of symptoms.

A recent study examined the expectations of 352 patients receiving total knee arthroplasty and showed that the fulfilment of their expectations was the basis for postoperative satisfaction [23]. As arthritis occurs more often in load-bearing joints, expectations with regard to knee and hip arthroplasty are generally better understood [24,25,26,27,28].

Studies examining expectations of patients with shoulder pathologies are still rare, especially with regard to total shoulder arthroplasty. The study by Mancuso et al. was one of the first to analyze patients’ expectations before a planned shoulder operation [11]. They demonstrated that patients’ expectations towards the outcome of surgery are dependent on diagnosis, demographics and functional status and developed a template for surgeons to discuss realistic and unrealistic goals with the patient and consequently improve shared decision making [11]. Kaveeshwar et al. interviewed 216 patients who underwent shoulder surgery, showing that greater expectations are predictive for better outcome scores with reference to pain relief, shoulder function and social satisfaction [29].

Regarding reverse shoulder arthroplasty (RSA), Lizzio et al. reported that lower preoperative satisfaction was associated with greater overall expectations for surgery [12]. Rauck et al. stated that patients undergoing RSA have high expectations for pain relief and simple task performance, especially if they have higher preoperative function [30]. Examining patients who underwent anatomic total shoulder arthroplasty (TSA), a study demonstrated that those patients had higher expectations for return to exercise compared with patients receiving reverse shoulder arthroplasty, which was positively correlated with postoperative functional outcome [31]. This outcome was confirmed by Swarup et al., who demonstrated a better outcome in patients with greater preoperative expectations [32].

Generally, patients planned for a shoulder operation have high preoperative expectations which should be discussed preoperatively to help develop realistic goals [33,34]. Different studies confirm that patients who know what to expect from surgery have an improved postoperative outcome [35,36,37,38,39]. One goal of the surgeon should therefore be to inform properly about the underlying pathology, planned operation and aimed outcome to improve satisfaction on both sides.

This study also has limitations. Patients’ might have set their queried expectations higher than they actually were, alarmed that lower expectations might lead to lower surgical effort and therefore a worse outcome. The possibility that some patients might have answered in favor of the surgeon could not be fully eliminated. Moreover, patients saw their surgeon during a consultation hour before undergoing surgery, which may also have introduced a certain bias. Furthermore, the number of patients included in the study was relatively low, although the results were in line with other studies.

## 5. Conclusions

Studies evaluating patients’ preoperative expectations are increasingly coming into focus. This study shows that patients’ expectations for shoulder joint replacement and surgeons’ assessment do not differ significantly. The most important goal to achieve for patients suffering from osteoarthritis was ‘to relieve daytime pain’, followed by ‘to improve self-care’ and ‘the ability to reach above shoulder level’. Younger patients were more willing to accept a longer period of immobilization, postoperative pain and aftercare. Regarding the most important factor for selecting the surgeon, ‘surgical skills’, was ranked in first place by patients, whereas surgeons thought that ‘age and experience’ would be the most important.

Expectations should be taken into account within the preoperative medical interview, as well as what to expect postoperatively with regard to outcome and necessary aftercare. This might allow an optimization of compliance of the patients and lead to better satisfaction on both sides.

## Figures and Tables

**Table 1:** The influence of patients’ demographics and preoperative scores on the expectations (* *p* < 0.05, ** *p* < 0.01, n.s. = not significant). A negative correlation means that the higher the data or score was (*y*-axis), the lower the rank was for the expectation (*x*-axis).

	Relieve Daytime Pain	Relieve Nighttime Pain	Participate in Sports	Be Employed for Monetary Reimbursement	Move-Ment	Scar	Immobili-zation	Physio-therapy	Pain	After-Care	Sympathy
age	n.s.	n.s.	0.402 **	n.s.	n.s.	n.s.	-0.332*	n.s.	−0.404 **	−0.313 *	n.s.
BMI	0.321 *	n.s.	n.s.	n.s.	n.s.	n.s.	n.s.	n.s.	0.322 *	n.s.	n.s.
ASES	n.s.	n.s.	n.s.	−0.305*	−0.281 *	−0.301 *	n.s.	n.s.	n.s.	n.s.	n.s.
SSV	n.s.	0.354 *	−0.295 *	n.s.	n.s.	n.s.	n.s.	n.s.	n.s.	n.s.	0.317 *
VAS	n.s.	−0.304 *	n.s.	n.s.	n.s.	n.s.	n.s.	−0.342 *	n.s.	n.s.	n.s.
duration of symptoms	n.s.	n.s.	n.s.	n.s.	n.s.	n.s.	n.s.	n.s.	n.s.	n.s.	0.371 **

## Data Availability

The original contributions presented in the study are included in the article, further inquiries can be directed to the corresponding author.
